# A hybrid ceramifiable hydrophobic coating with integrated fire-resistance and weatherability

**DOI:** 10.1039/d6ra08072c

**Published:** 2026-10-06

**Authors:** Junhui Zhang, Guanyuan Zheng, Haichun Zhang, Xiru Zhang, Baoku Sun, Ading Lu, Huisheng Fan, Dandan Jin, Lei Li

**Affiliations:** a School of Aeronautics, Shanghai Dianji University Shanghai 201306 China 841064154@qq.com; b Zhejiang Marine Development Research Institute Zhoushan Zhejiang 316021 China lilei@sdju.edu.cn

## Abstract

Steel structures in coastal oil and gas facilities are particularly susceptible to corrosion and fire hazards, posing significant challenges to structural integrity and fire safety. Anti-corrosion coatings provide excellent corrosion resistance for the steel, while, in the case of the 1200 °C flame conditions, inorganic passive fire protection coating provides superior to fire performance than the organic coating. Here, a hybrid ceramifiable hydrophobic coating was developed by incorporating a high loading of two-dimensional flake fillers into a silicone resin matrix to enhance fire resistance of organic anticorrosion-coated structural steel. The results indicate that the developed coating exhibits excellent fire-resistance and weatherability. Under a 1200 °C flame, the fire-resistant coating protected the steel plate for at least 30 minutes without peeling, while limiting the backside temperature to approximately 535 °C. Moreover, the weatherability of the coating was assessed *via* accelerated environmental aging, including corrosive and damp-heat environments. This excellent stability is attributed to the dense packing of the flake fillers and the inherent hydrophobicity of the silicone resin. These findings not only provide condensed-phase fire protection but also serve as a candidate for the potential future applications in diverse extreme service conditions, such as lithium-ion battery enclosures.

## Abstract 2

Steel structures in coastal oil and gas facilities are particularly susceptible to fire hazards and corrosion, posing significant challenges to fire safety and structural integrity.

## Graphical abstract

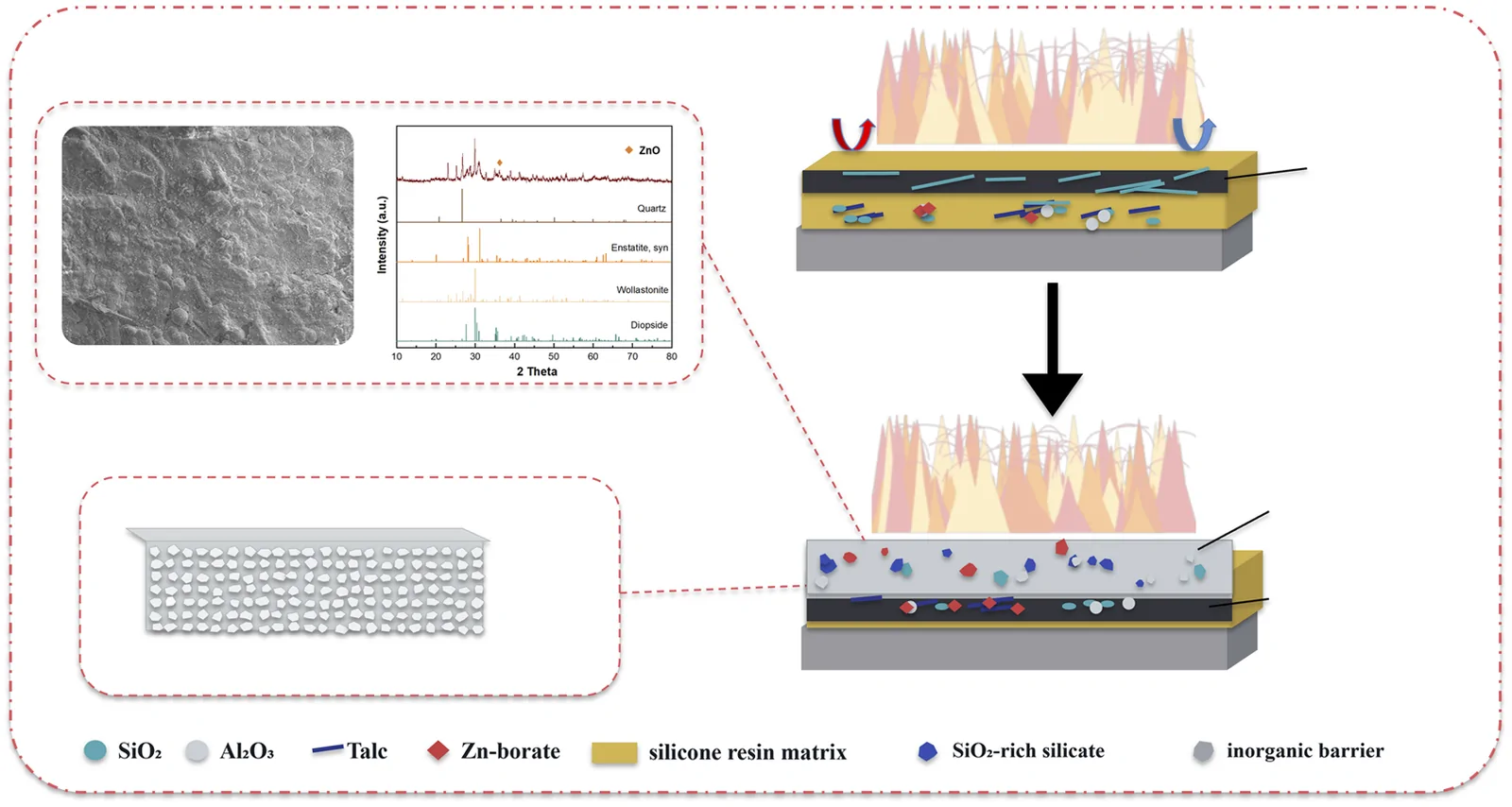

## Introduction

Steel structures are widely used in oil and gas facilities owing to their high strength and ease of fabrication. However, their high thermal conductivity and the rapid deterioration of their mechanical properties at temperatures above 600 °C make them highly vulnerable to fire, potentially resulting in structural instability and catastrophic safety failures. Consequently, the application of fire-resistant coatings has become one of the most effective approaches for enhancing the fire resistance of steel structures.^1–5^

These coatings are generally classified into non-intumescent and intumescent types. Non-intumescent fire-resistant coatings mainly provide thermal insulation through their inherent thickness, and those are typically formulated with inorganic binders, such as silicate cement or gypsum-based materials, exhibit poor adhesion to these primers. Intumescent fire-resistant coatings, in contrast, use polymer binders, flame-retardant additives and fillers to form an expanded char layer that limits oxygen access and heat transfer. They expand rapidly at elevated temperatures, forming a dense carbonized insulation layer that effectively blocks oxygen and thermal radiation.^6–11^ However, the resin matrix (polymer binders) is highly combustible and easily undergoes pyrolysis at high temperatures. At temperatures above 900 °C, the carbonaceous char layer undergoes progressive oxidation and decomposition, which may ultimately lead to its complete disappearance and a significant loss of fire resistance.^12–15^ The loss of the protective char residue compromises the fire resistance of the coating and is typically accompanied by heat release, smoke generation, and the release of toxic volatile products.

Fire safety remains a persistent concern in oil and gas facilities because fires can develop rapidly and release high heat fluxes. Depending on their characteristics and fuel-release conditions, such fires are commonly described as standard fires, pool fires and jet fires. In the event of standard fires, the exposed side temperature of about 1000 °C is slowly obtained; in the instance of a pool fire, the exposed side temperature of about 1095 °C is attained and maintained throughout fire exposure; meanwhile, jet flames may reach temperatures close to 1200 °C and heat fluxes up to about 300 kW m^−2^.^16–18^ Among them, the worst-case scenario is jet fire. At such elevated temperatures, not only does the organic char layer formed by conventional intumescent systems undergo oxidation and decomposition, but some inorganic constituents may also melt or decompose.^19–24^ In recent years, ceramifiable polymer materials have therefore attracted considerable attention. These materials behave like conventional polymers at ambient temperature. During combustion, fluxing-agent fillers embedded in the polymer matrix melt to form a continuous ceramic or glass phase that bonds refractory fillers and the carbonaceous residue from the polymer. The composites undergo a ceramification reaction at high temperatures, rapidly transforming into a mechanically robust ceramic structure. Ceramifiable flame-retardant technology can markedly enhance both the fire resistance and the mechanical strength of the protective layer.^24–31^ It combines the processability and mechanical flexibility of polymers with the high-temperature stability of ceramics. He *et al.*^32^ have designed several ceramifiable EVA composites, which are further utilized as barriers to suppress TRP of nickel-cobalt-manganese batteries. Reported ceramifiable EVA composites for suppressing thermal runaway propagation in batteries, with char residue increasing from 0.15% for neat EVA to 35.2%. However, excessive or rapid ceramization can increase the hardness and brittleness of the protective layer, thereby promoting crack propagation, and eventual exposure of the substrate under severe flame conditions. In addition, low-melting glass phases may undergo excessive softening or melting at temperatures above 1000 °C, which can compromise the structural integrity of the protective layer. Although the coating combining intumescent and ceramifiable systems exhibit distinct advantages in thermal insulation and fire resistance, the use of hydrophilic phosphate compounds such as ammonium polyphosphate possibly can reduce the hydrophobicity and long-term weather resistance of the coating.^33–35^

A large number of petrochemical facilities are intensively deployed in coastal areas mainly due to the superior logistics capacity of deep-water seaports, to mitigate risks of hydrocarbon fire and explosion, and to cut energy consumption. However, coastal high-humidity and high-salinity environments seriously challenge the durability of steel structural members. It remains a great challenge to develop coatings with both excellent fire resistance and anti-corrosion performance for coastal facilities. The most cost-effective scheme consists of priming the steel substrate with an organic anti-corrosion coating prior to the top-coating of fire-resistant coating.^36^ Moreover, few studies have been reported on the hydrophobicity and long-term weather resistance of the ceramifiable fire-protective coatings.

To overcome the above challenges, we developed an organic–inorganic hybrid coating with ceramization behavior, which enables the formation of a structurally stable protective layer at high temperatures and provides effective protection against flame ablation at 1200 °C. After ablation, a protective ceramic layer was generated on the exposed surface, with only slight microcracks present. Wherein, silicone resin was selected as the binder because it can generate silica-rich products during high-temperature oxidation; however, the pyrolysed silica alone tends to form a weak, powdery residue near 1200 °C. Refractory fillers and ceramifying additives are therefore introduced to build a more coherent protective layer. The chemical structure, morphology, and composition of the composite coatings have been identified and analyzed by Fourier transform infrared spectroscopy (FTIR), energy dispersive X-ray spectroscopy (EDS), scanning electron microscopy (SEM), respectively.

To evaluate the thermal-protection capability and structural integrity of the coating under severe direct flame exposure, the fire-protective performance was assessed using a butane-fueled Bunsen burner, which generates a flame temperature of approximately 1200 °C. The results indicate that the coating exhibited excellent fire resistance, and the underlying fire-protection mechanisms were proposed based on an analysis of the residues collected after the fire tests. Meanwhile, integrate hydrophobicity has been investigated in detail, enabling durable multifunctional protection of outdoor steel structures under aggressive service environments. Overall, this work has offered a convenient and effective strategy to solve the intractable difficulty in the traditional coatings for steel structures and is expected to further promote its application value in important practical fields such as construction, automotive engineering, and aerospace.

## Materials

Magnesium silicate monohydrate (Talc, D50 11.22, Oil absorption value 35–50 g/100 g) is purchased from Aladdin. SiO_2_ (purity 99.81 wt%, D50 4.81 µm, specific surface area 2.0 m^2^ g^−1^) and Al_2_O_3_ (purity 99.81 wt%, D50 5.80 µm, specific surface area 0.6 m^2^ g^−1^) are obtained from NOVORAY Staff. Silicone resin (liquid, Viscosity@25 °C 2–20 mPa s) and curing agent are provided by Wynca Group. All other reagents are analytical grade. Silicone resin and supporting curing agent are mixed with a 20 : 1 mass ratio as coating binder.

### Preparation of coatings

Talc, SiO_2_, Al_2_O_3_ and silicone resin were mixed until a homogeneous dispersion was obtained. Additives, including a defoamer and a ceramifying additive (including 4ZnO B_2_O_3_·H_2_O, α-CaSiO_3_), were subsequently introduced and stirred for 10 min. A small amount of curing agent was added in the final stage to avoid premature curing while maintaining uniform dispersion. The slurry was applied to different substrates for subsequent characterisation. The coated specimens were kept at room temperature for 0.5 h to achieve surface drying and for 24 h for full drying, as shown in Fig. 1. The sample formulations are listed in Table 1.

**Fig. 1:**
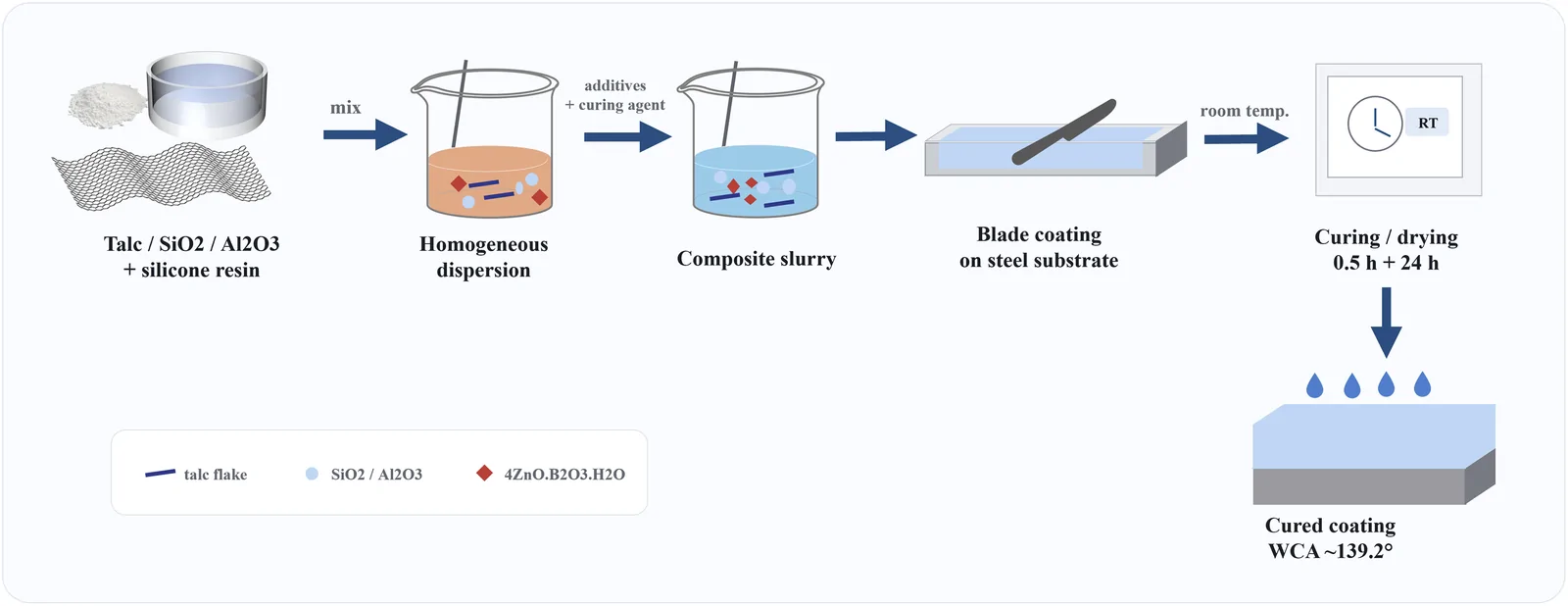
Preparation process of the organic–inorganic hydrophobic fire-resistant coating.

**Table 1:** The formulation of the coating

No.	Silicone resin (phr)	Additive (phr)	SiO_2_ (phr)	Al_2_O_3_ (phr)	Talc (phr)
C-0	10	5	45	2	0
C-20	10	5	25	2	20
C-30	10	5	15	2	30
C-40	10	5	5	2	40
C-45	10	5	0	2	45

### Characterization

The crystalline structure and chemical composition of the prepared coatings and substrates were characterized by Fourier transform infrared (FT-IR) spectroscopy using an attenuated total reflection (ATR) method. FT-IR spectra were recorded on a Cary 660/620 spectrometer (Agilent, USA) over a wavenumber range of 400–4000 cm^−1^ with a resolution of 4 cm^−1^.

The microscopic morphologies of the fire-resistant coating and char residues were conducted by a JEOL JSM-6500F field emission scanning electron microscope (FE-SEM) equipped with an energy-dispersive X-ray spectrometer (EDS, Oxford Instruments, UK).

The crystalline phases of the fire-resistant coating were determined using an X-ray diffractometer (XRD, Bruker D8 Advance, Germany) with Cu Kα radiation (15 mA and 40 kV) with scan rate 0.1 s/°.

The adhesion between the fire-resistant coating and the substrates was tested using an adhesion tester according to the procedure of ASTM D3359-09. Adhesion was classified on a scale from 0B to 5B.

To evaluate the protective performance, steel plates were coated with a 2 mm-thick layer and subjected to fire-resistance testing. The thickness of the fire specimen was checked with thickness gauge, make sure that the dry coating thickness was strictly controlled within 2.0 ± 0.2 mm. A butane-fueled Bunsen burner was applied directly to the coated surface, maintaining a nozzle-to-plate distance of 4 cm and a flame temperature of approximately 1200 ± 100 °C for 30 minutes. Surface temperatures on both the exposed (hot) and unexposed (cold) sides of the steel plates were measured using Fluke RSE30H and Fluke Ti401U thermal imagers (Fluke, USA), respectively. Three independent replicate specimens were tested under the same experimental condition to demonstrate experimental reproducibility. For a conservative assessment, the profile corresponding to the highest backside temperature was selected for the subsequent discussion.

Furnace tests were conducted to analyze the thermal performance of the coating. The samples were combusted in a muffle furnace at a heating rate of 20 °C min^−1^ until they reached 400 °C, 600 °C, 800 °C, 1000 °C and 1200 °C and maintained for 20 min. This ensured the coatings were completely combusted. The mass change before and after the test as well as the sample state were recorded.

The wettability of the fire-resistant coating was investigated by contact angle (model 190-F2, Rame-hart CA instrument) measurements using the sessile drop method with 5 µL deionized water droplets. The contact angle measurements were taken from three different points on the surface of each sample, and the values closest to the average were recorded.

## Result and discussion

### Optimization of the organic–inorganic coating formulation

To fabricate a ceramifiable fire-resistant coating with a high filler loading, three major constituents are normally required: (1) a silicone resin, which serves as both the coating matrix and the binder for the other components; (2) ceramic fillers, which are inherently nonflammable and function as thermal-shielding fillers; and (3) low-content additives, which enhance filler dispersion and contribute to the development of a ceramic network, functioning as a protective fire barrier under high-temperature conditions. The ceramic fillers used typically include silicon dioxide powder, alumina powder, and talc. Silicon dioxide powder exhibits weak interfacial bonding and limited mechanical strength; alumina powder is incorporated to promote the formation of a ceramic network at high temperature, and talc is introduced to enhance the mechanical strength of the coating.

As shown in Fig. 2, for C-0, which does not contain talc, wide and deep mud-crack patterns are observed on the coating surface. As the talc content increases, these mud cracks gradually become finer and less frequent. Ultimately, sample C-40 exhibits a smooth and crack-free surface, which can be attributed to the bridging effect of the talc particles. The cracks reappear on the coating surface once more with the increase of the talc (C-45), due to the markedly higher oil absorption of the layered structure compared to the granular counterpart.

**Fig. 2:**
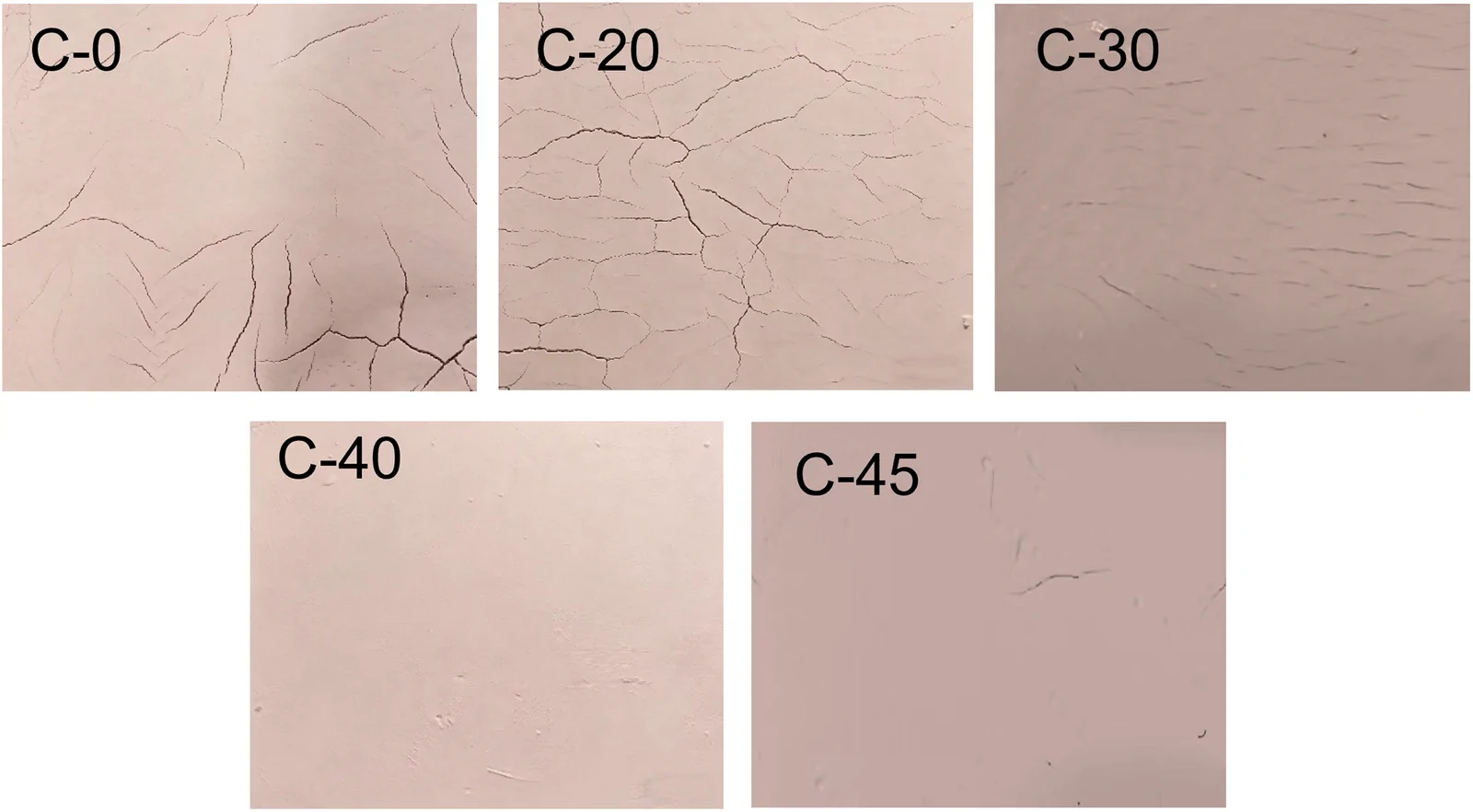
Digital photos of the coating surface with different contents of talc.

To demonstrate the formation of the fire-resistant coating, Fourier transform infrared (FT-IR) analysis were conducted on the sample to probe molecular vibrations/functional groups for the sample with 40wt% talc, while X-ray diffraction (XRD) analysis was conducted to provide crystal structure. As shown in Fig. 3a, the characteristic peaks at 1272 and 782 cm^−1^ are attributed to the symmetric deformation and rocking vibrations of Si–CH_3_ groups, respectively, confirming the presence of the silicone resin. While the Si–O–Si stretching vibration and M–O (M: Mg, Al) stretching vibration appearing at the ∼1006 cm^−1^ and ∼668 cm^−1^, respectively, are from inorganic components. As shown in the XRD pattern (Fig. 3b), the peaks at 9.6°, 19.0°, 28.7°, and 48.7° closely match Talc; The broad diffraction halo centered at approximately 12.1° is attributed to the amorphous silicone resin phase; The peaks at 23.2°, 25.3° and 26.9°correspond to 4ZnO B_2_O_3_·H_2_O, α-Al_2_O_3_ and crystalline quartz SiO_2_, respectively. The EDS analysis (Fig. 3c) on the surface of the coating shows that the major elements are Si and O, and the second constituent elements are Mg and Ca. According to the mapping results, Si, O and Mg are uniformly dispersed.

**Fig. 3:**
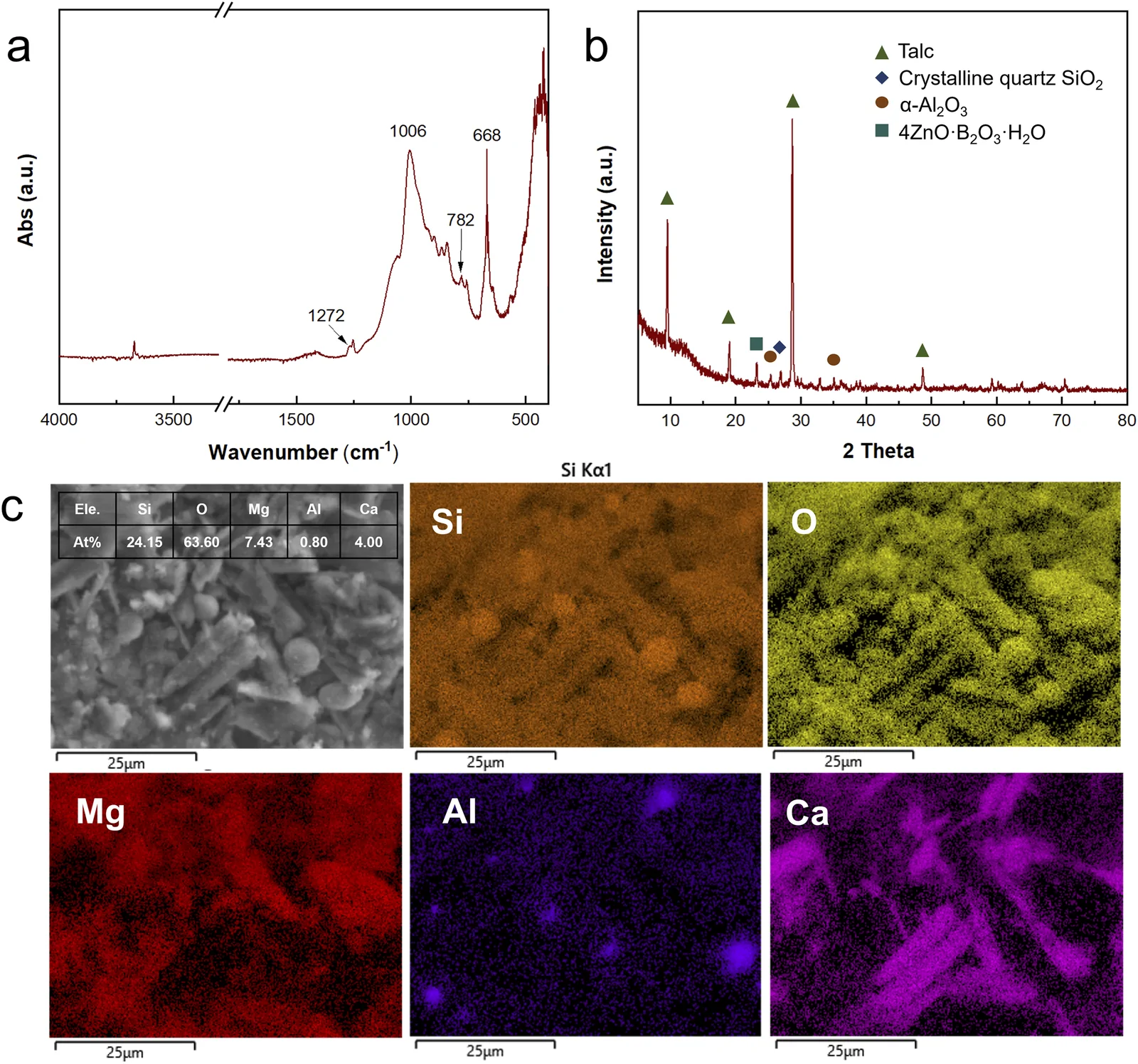
(a) The FTIR spectra and (b) the XRD pattern of the coating; (c) the EDS elemental mapping of Si, O, Mg, Al and Ca for the fire-resistant coating.

In summary, the XRD results are consistent with the FTIR findings, confirming the successful formation of the coating system.

### The adhesion of the coating

Strong adhesion between the coating and substrate is essential for maintaining the integrity and protective performance of the coating under service conditions. Therefore, the adhesion grades of the coatings on different substrates were evaluated, and the results are presented in Fig. 4.

**Fig. 4:**
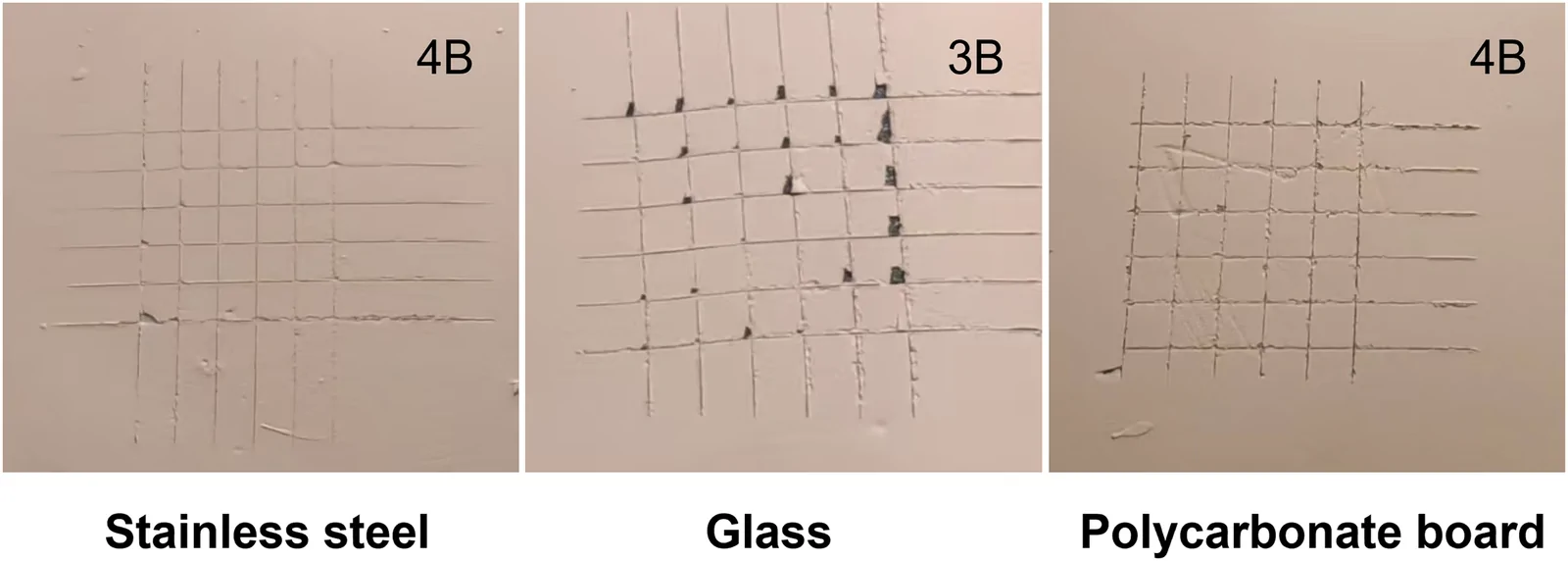
Digital photos of the adhesion grade of the coatings after the adhesion testing on the different substrates.

A 5B rating corresponds to the highest level of adhesion, whereas a 0B rating represents the poorest adhesion. The adhesion performance varies depending on the substrate type. The coating on stainless steel and on the polycarbonate board exhibits a detachment area of approximately 3%, corresponding to a 4B adhesion rating, whereas the coating on glass shows a detachment area of approximately 13% and only a 3B adhesion rating. Therefore, on the stainless steel and polycarbonate substrates, the adhesion meets the acceptance criteria.

### Fire performance of the coating

Owing to its excellent load-bearing capacity, steel is widely used in the construction of high-rise and super high-rise buildings. However, its mechanical properties are highly sensitive to temperature; once exposed to elevated temperatures, its strength deteriorates rapidly, which can lead to sudden instability or even collapse of steel structures during a fire. Therefore, the thermal insulation and fire-protection performance of the organic–inorganic hydrophobic coating on stainless steel substrates were further evaluated. However, the test has inherent limitations. In particular, the butane-fueled Bunsen burner test used in this study should not be considered directly equivalent to a standardized jet-fire test.

Fig. 5a and b show stainless steel substrates with and without the fire-resistant coating subjected to a flame at around 1200 °C. While Fig. 5d presents the temperature–time curves on the unexposed (cold) surface of stainless-steel substrates with and without the fire-resistant coating during the fire-resistance test. The backside temperature of the bare steel plates rises rapidly to approximately 700 °C within 5 min of flame exposure, which will pose a serious risk of structural collapse and a significant threat to human life and safety in the event of an uncontrolled fire. In contrast, upon flame exposure, the central temperature on the backside of the coated steel plate increases gradually from room temperature to approximately 400 °C within 5 min and then continues to rise to 535 °C within 30 min, owing to the good fire and thermal inhibition action of the coating. Stainless-steel substrates with the fire-resistant coating after the fire-resistance test were investigated, as shown in Fig. 5c. The coating in the flame-exposed area appears grayish white and exhibits fine cracks, indicating the formation of a protective ceramic-like layer during fire exposure. In contrast, no obvious morphological changes are observed in the coating outside the flame-exposed area for visual inspection.

**Fig. 5:**
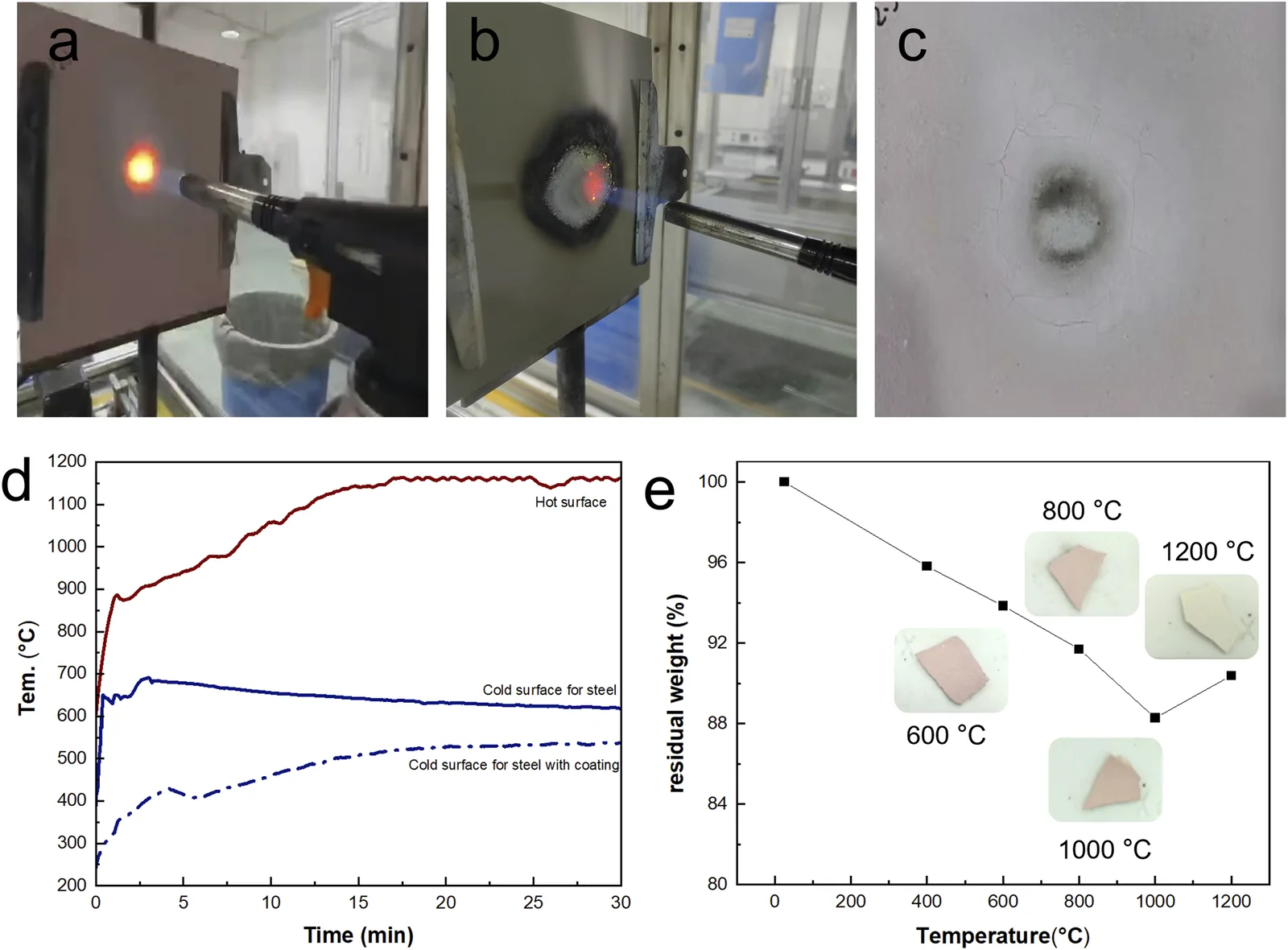
Digital photos of stainless-steel substrates (a) with and (b) without the fire-resistant coating during the fire-resistance test; (c) the digital photo of coating after fire test; (d) the temperature–time curves on the exposed (hot) and unexposed (cold) surface; (e) the residual weight-temperature curve recorded by the furnace test and the sample morphology of the fire-resistant coating after furnace tests.

Fig. 5e shows the residual weight percentage *versus* temperature and the sample morphology after furnace tests for the fire-resistant coatings exposed to the heating rates 20 °C min^−1^. An initial period of mass loss was observed between 200 and 1000 °C, and the sample remains pink throughout the process; then, a slight increase in mass due to the oxidative conditions between 1000 and 1200 °C, and the sample turned white after heating at 1200 °C. Furthermore, a minimum residual mass of 88.28% is maintained during the test, due to the high inorganic filler loading.

The surface morphologies of the organic–inorganic fire-resistant coating are shown in Fig. 6b and c, corresponding to the unexposed and exposed flame-exposed areas, respectively. The unexposed area exhibits a relatively uniform surface morphology, with flake-like fillers densely and orderly distributed on the coating surface. In contrast, the flame-exposed area presents a continuous and compact surface covered by particles, which are tentatively attributed to metal and non-metal oxides. Moreover, EDS analysis of the char-layer surface (Fig. 6d) reveals a pronounced enrichment of Si, accompanied by a substantial decrease in the Mg and Ca contents. This compositional evolution suggests that Si-containing particles migrated toward the coating surface under flame exposure and subsequently underwent oxidation, contributing to the formation of a protective Si-rich oxide.

**Fig. 6:**
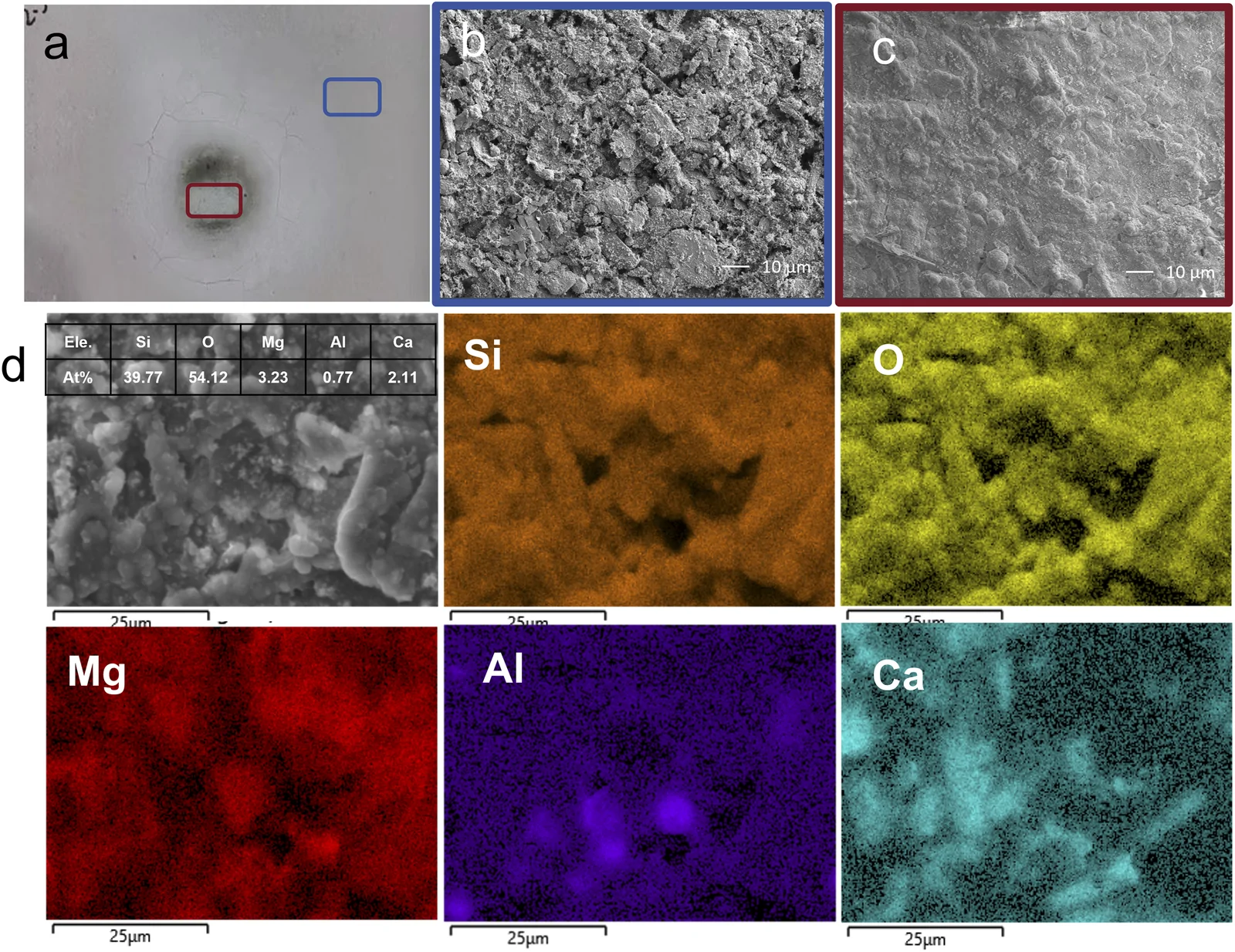
(a) Photograph of the fire-protective coating after the flame-exposure test. SEM images of the (b) unexposed area and (c) flame-exposed area of the coating. (d) EDS elemental mapping of Si, O, Mg, Al, and Ca in the flame-exposed area.

According to the results of the FT-IR analysis shown in Fig. 7a, except for the peaks at ∼1006 cm^−1^ and ∼668 cm^−1^, no other peaks are found, further indicating that organic compounds convert to inorganic compounds *via* pyrolysis. XRD can provide complementary evidence of phase transformations within the protective residue;^37^ Obvious structural and phase changes occurred in the coating after fire exposure, as shown in Fig. 7b. The characteristic peak at 9.6°, 19.0°, 28.7° of the talc disappeared, the new peaks at 26.7° and 28.0°/31.1° corresponding to quartz (SiO_2_, PDF 00-0046-1045), enstatite (Syn, Mg_2_(Si_2_O_6_), PDF 00-0019-0768) are observed; meanwhile additional distinct peaks at 23.1°, 25.2°, 26.8° and 30.0° assigned to wollastonite (CaSiO_3_), PDF 00-0027-0088) are identified, revealing that amorphous calcium silicate undergoes structural transformation. Further, new peaks at 27.7°, 30.0°, and 35.3° were assigned to diopside (Ca(Mg,Al)(Si,Al)_2_O_6_, PDF 00-0041-1370), suggesting the formation of this phase through high-temperature reactions involving the calcium- and magnesium-containing silicate phases. Other slight structural alterations can also be detected, wherein 4ZnO B_2_O_3_·H_2_O undergoes thermal decomposition, generating zinc oxide alongside other phases.

**Fig. 7:**
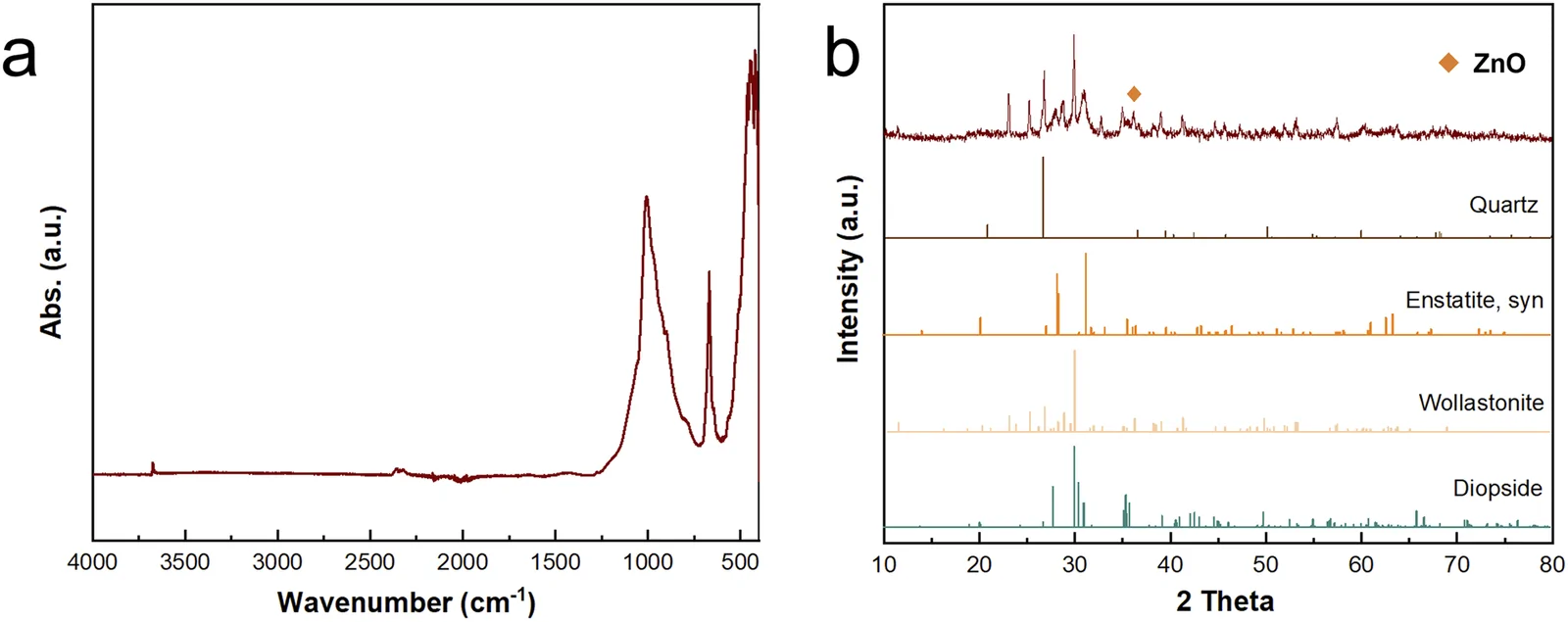
(a) FTIR spectra and (b) XRD patterns of combustion coating.

Collectively, these results demonstrate that the thermal decomposition of the silicone resin and the high-temperature transformation of the inorganic phases occurred simultaneously, contributing to the formation of a crystalline ceramic-like protective residue.

In conclusion, as illustrated in Fig. 8, the proposed fire-protection mechanism of the silicone-based coating involves the initial thermal decomposition of the silicone resin, accompanied by the formation of silicone oligomers and carbonaceous species. These pyrolysis products subsequently migrate toward and redeposit on the char-layer surface, where they undergo further oxidation and ceramification, leading to the formation of Si-containing particles and silica (SiO_2_). This process ultimately produces a porous yet mechanically robust ceramic protective layer that effectively retards heat transfer and protects the underlying substrate from flame exposure.^38,39^

**Fig. 8:**
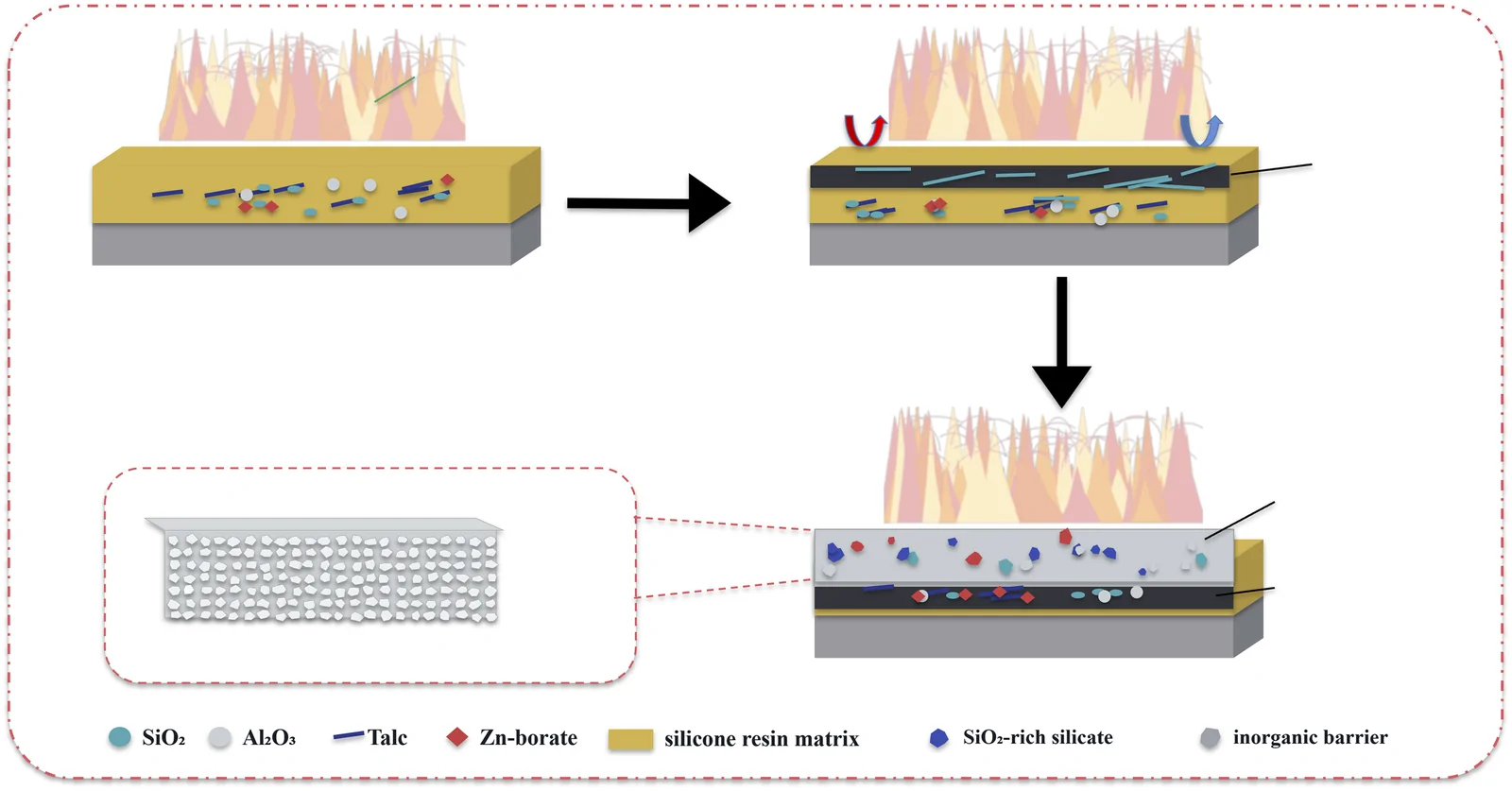
Schematic illustration of inorganic compound barrier formation during flame exposure.

### The weatherability of the coating

The complex service environments of multiscale fire-protective coating demand excellent water resistance to ensure long-term protective performance.^40^ Enhanced water resistance is particularly important for ensuring the long-term reliability of these coatings in outdoor environments. Therefore, methyl groups were introduced into the silicone resin to modify the coating surface and enhance its hydrophobicity.

As shown in Fig. 9a, the pristine sample exhibited a water contact angle (WCA) of 139.2°, confirming its intrinsic high hydrophobicity. Furthermore, accelerated aging tests were conducted to simulate and accelerate the degradation processes of the coatings under harsh environmental conditions, thereby assessing their long-term durability and retention of hydrophobic functionality. Under prolonged environmental exposure, the metastable, surface-enriched hydrophobic layer may be disrupted, exposing the underlying bulk material and consequently reducing surface hydrophobicity. Accordingly, the effects of different aging environments on the coating were systematically evaluated by visual inspection and water contact angle measurements after water immersion, 85 °C/85% RH aging, and neutral salt spray exposure. As shown in Fig. 9, visual inspection revealed no apparent coating defects, including whitening, blistering, gloss loss, cracking, rust formation, or delamination, after 24 h of water immersion at room temperature, 500 h of 85 °C/85% RH aging, and 250 h of neutral salt spray exposure. The absence of visible deterioration suggests that the coating possesses good resistance to environmental aging and corrosion.

**Fig. 9:**
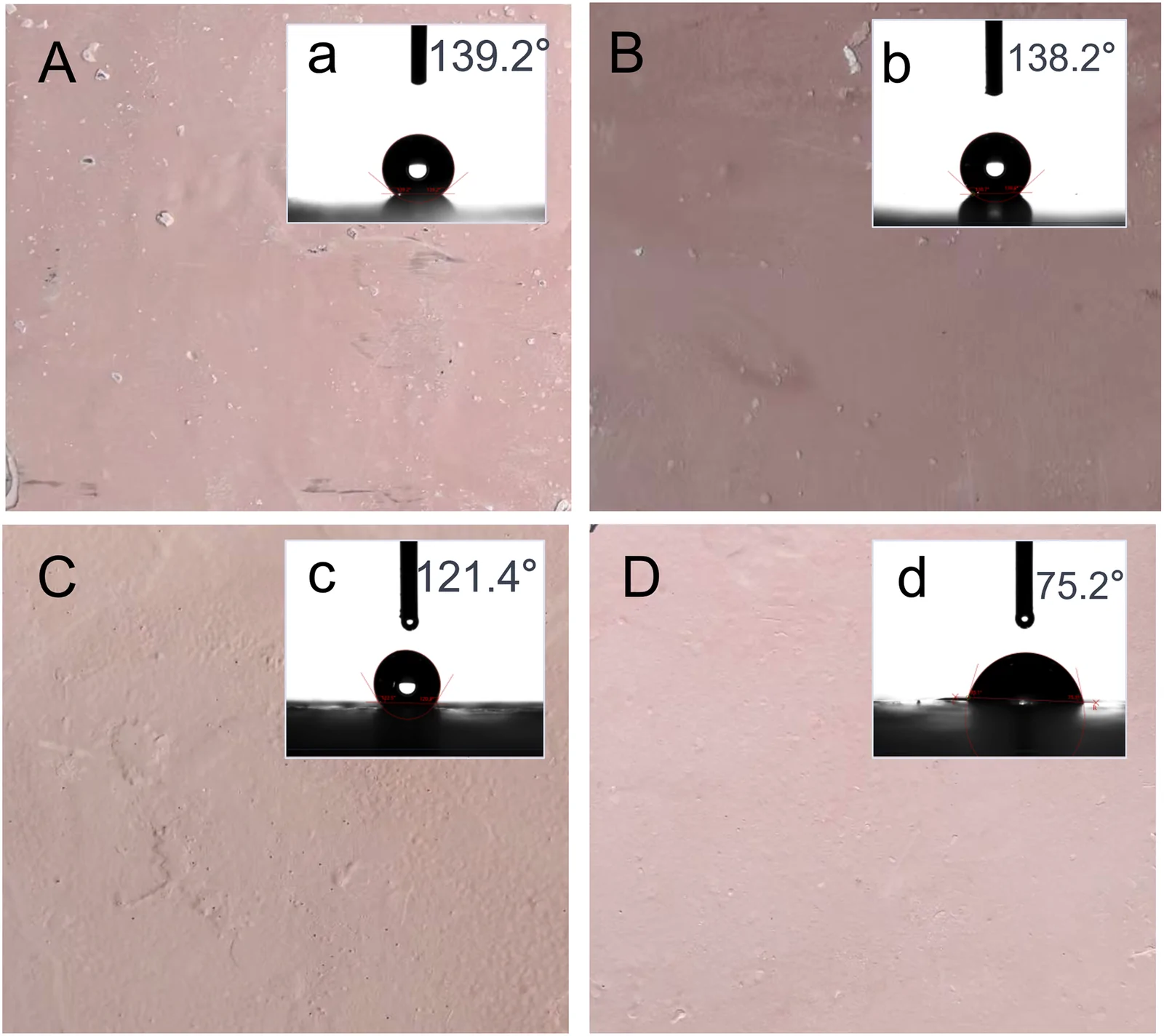
The digital photos and the WCA images (inserted) of the coating after different processing methods: (A, a) the pristine sample, (B, b) after soaking in water at room temperature for 24 h, (C, c) after 500 h of double-85 aging, (D, d) after 250 h of neutral salt spray aging.

As strong water–surface interactions may induce surface reorganization. While contact angle testing can amplify such subtle surface deterioration more obviously than visual observation. After soaking in water at room temperature for 24 h, the contact angle of the coating remains at 138.2° (shown in Fig. 9b), indicating that the coating is chemically stable, dense, and durable in aqueous environments, which is critical for long-term performance. As shown in Fig. 9c, after 500 h of 85 °C/85% RH aging, the contact angle decreases to 121.4°, corresponding to a reduction of 17.8° compared with the unaged sample, indicating that chemical and/or microstructural changes occurred on the material surface. Based on the above results, it can be concluded that the coating possesses certain hydrophobic stability when exposed to high temperature and high humidity. Meanwhile, after 250 h of neutral salt spray aging, the water contact angle decreases to 75.2° (Fig. 9d), representing a substantial decline relative to the unaged sample and indicating a pronounced loss of surface hydrophobicity under saline conditions.

To analyze the changes in the contact angle of the coating surface after different treatments, SEM was used to characterize the microscopic changes on the coating surface. As shown in Fig. 10a and b, the surfaces of the pristine sample and the sample soaked in water for 24 h remain relatively smooth and dense. In contrast, the samples subjected to double-85 aging and neutral salt spray aging exhibit noticeably loose and more disordered morphologies (Fig. 10c and d). This surface degradation directly correlates with the observed decrease in contact angle. Elemental mapping results (Fig. 10e and f) further confirmed the non-uniform surface distribution of Si and Mg after aging treatment. The coatings were further characterized by FT-IR spectroscopy to explore the root causes. As depicted in Fig. 10g, after soaking in water at room temperature for 24 h, the peak intensity of the Si–O–Si stretching vibration (∼1006 cm^−1^) is obviously enhanced, compared to the M–O (M: Mg, Al) stretching vibration (∼668 cm^−1^). Meanwhile, for the samples after 500 h of 85 °C/85% RH aging and 250 h of neutral salt spray aging, not only the similar phenomenon is observed, but also the peak intensity of the Si–CH_3_ symmetric deformation vibration and rocking vibration appearing at the 1272 and 782 cm^−1^ is obviously decreased. These results suggest that the aging process induces a chemical transformation within the silicone resin, characterized by the gradual consumption of methyl groups and the concurrent formation of additional Si–O–Si networks. Consequently, the deterioration of the coating's hydrophobicity during salt-spray exposure is associated with the disruption of the surface morphology and the reduction of hydrophobic methyl groups. These changes may result from degradation of the resin matrix during prolonged exposure to the high-humidity, high-salt environment, which can alter the surface morphology and reduce the availability of hydrophobic groups at the coating surface.

**Fig. 10:**
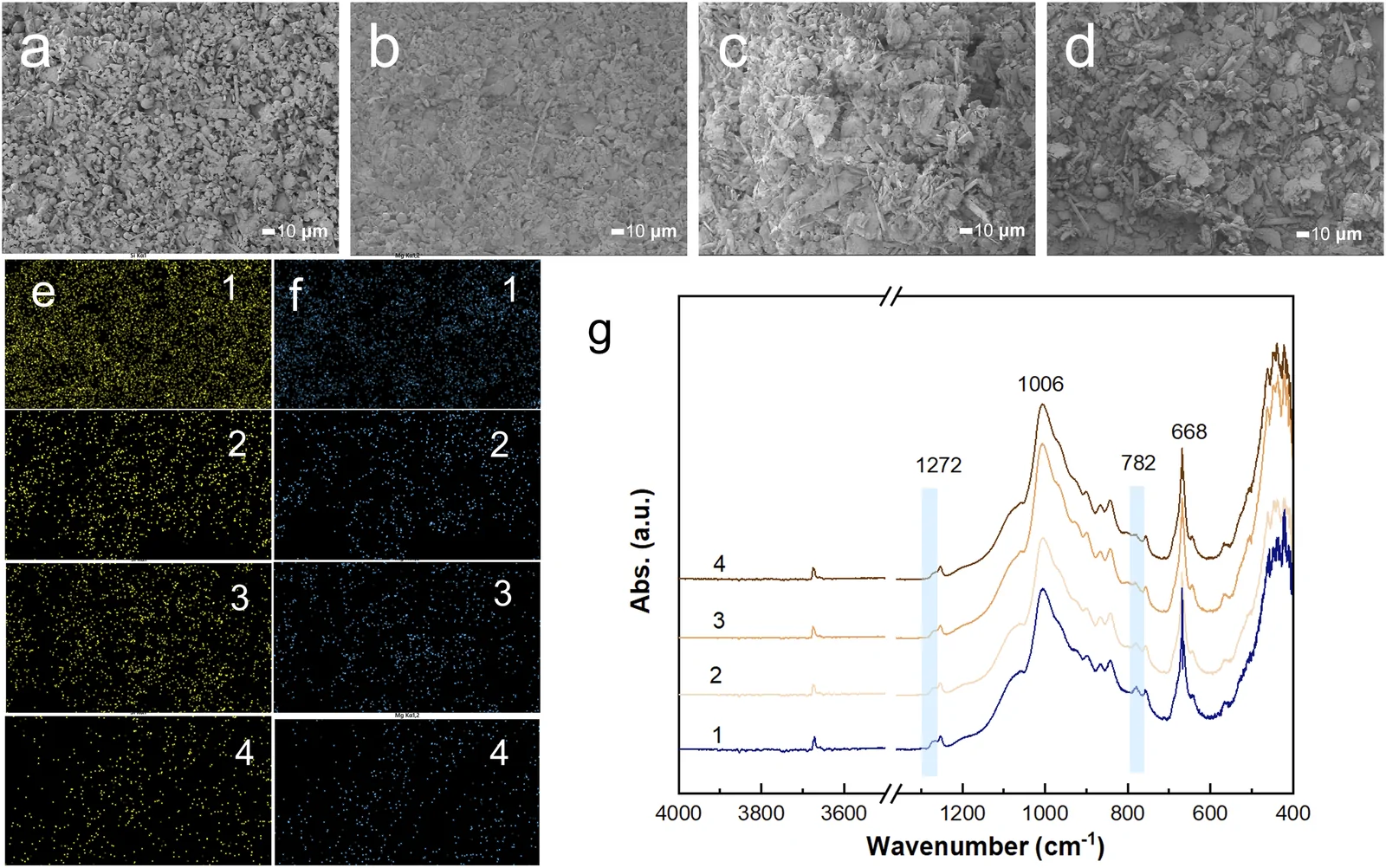
The SEM images of the coating after different processing methods: (a) the pristine sample, (b) after soaking in water at room temperature for 24 h, (c) after 500 h of 85 °C/85% RH aging, (d) after 250 h of neutral salt spray aging. EDS mapping of (e) Si and (f) Mg elements and (g) the FTIR spectra of the coating: (1) the pristine sample, (2) after soaking in water at room temperature for 24 h, (3) after 500 h of 85 °C/85% RH aging, (4) after 250 h of neutral salt spray aging.

## Conclusion

In summary, a ceramifiable organic–inorganic hybrid coating with high hydrophobicity and integrated fire-protection capabilities was successfully developed. FTIR, XRD, and EDS analyses confirmed the successful formation and structural characteristics of the designed organic–inorganic coating system. The coating exhibited multifunctional performance, including good adhesion, excellent fire resistance at elevated temperatures, and weatherability. Under direct flame exposure at approximately 1200 °C, the backside temperature of the steel plate coated with a 2 mm thick fire-protective layer gradually increased from room temperature to approximately 400 °C within 5 min and subsequently reached 535 °C after 30 min. Analysis of the post-fire residues suggested that the fire-protection mechanism primarily operated in the condensed phase, where the silicone resin-derived species redeposited and underwent further transformation to form a protective char/ceramic layer during heating. Moreover, the coating retained a relatively high degree of surface hydrophobicity after 24 h of water immersion and 500 h of aging at 85 °C/85% RH, with the WCA decreasing from 139.2° to 138.2° and 121.4°, respectively. However, prolonged salt-spray exposure resulted in a pronounced decrease in WCA to 75.2°, indicating limited hydrophobic durability under saline conditions. These findings demonstrate the potential of ceramifiable organic–inorganic hybrid coatings as multifunctional protective coatings that combine fire resistance, hydrophobicity, and environmental durability for structural steel applications.

## Conflicts of interest

There are no conflicts to declare.

## Data Availability

Data will be made available on request.
